# Stress Recovery during Exposure to Nature Sound and Environmental Noise

**DOI:** 10.3390/ijerph7031036

**Published:** 2010-03-11

**Authors:** Jesper J Alvarsson, Stefan Wiens, Mats E Nilsson

**Affiliations:** Gösta Ekman Laboratory, Department of Psychology, Stockholm University, SE-106 91 Stockholm, Sweden; E-Mails: sws@psychology.su.se (S.W.); mats.nilsson@psychology.su.se (M.E.N.)

**Keywords:** soundscape, nature sounds, environmental noise, skin conductance level, heart rate variability, stress recovery

## Abstract

Research suggests that visual impressions of natural compared with urban environments facilitate recovery after psychological stress. To test whether auditory stimulation has similar effects, 40 subjects were exposed to sounds from nature or noisy environments after a stressful mental arithmetic task. Skin conductance level (SCL) was used to index sympathetic activation, and high frequency heart rate variability (HF HRV) was used to index parasympathetic activation. Although HF HRV showed no effects, SCL recovery tended to be faster during natural sound than noisy environments. These results suggest that nature sounds facilitate recovery from sympathetic activation after a psychological stressor.

## 1. Introduction

In 1984, Ulrich demonstrated that patients whose windows faced a park recovered faster compared with patients whose windows faced a brick wall [1]. Since then, several studies have demonstrated restorative effects of natural compared with urban environments; these effects include increased well-being, decreased negative affect and decreased physiological stress responses [2–7]. Ulrich [6] suggested that natural environments have restorative effects by inducing positive emotional states, decreased physiological activity and sustained attention. This agrees with Kaplan and Kaplan’s theory that nature environments facilitate recovery of directed attention capacity and thereby reducing mental fatigue [8], and with results showing that positive emotions improves physiological recovery after stress [9].

Previous research in this area has mainly used visual stimuli, for example videos and photographs of nature settings and urban areas [1,5,10]. However, sound stimulation is also known to be a potent stressor, evoking unpleasant feelings (annoyance) and physiological stress reactions, especially at high sound pressure levels [11,12]. Studies on the connection between sound environment and stress recovery are currently lacking. Soundscape research has shown that natural sounds are typically perceived as pleasant and technological noise as unpleasant components of the sound environment [13–15]. It is therefore plausible that the sound environment may have a similar effect on stress recovery as the visual environment.

Ulrich *et al*. [6] used video films with sound and found faster physiological stress recovery during exposure to films depicting nature compared with urban environments. However, Ulrich *et al.* did not control for sound pressure level. Indeed, the soundtrack to their films of urban environmental settings had considerably higher sound pressure levels than the soundtrack to the films of nature environments. This makes it difficult to determine whether the effect was related to the characteristics of the environments or to differences in sound pressure levels. So, although positive effects of visual natural environments are well established, no research has been done using only auditory stimulation with controlled stimuli and sound pressure levels.

The autonomic nervous system controls various body functions: the sympathetic branch primarily controls activation and mobilization, and the parasympathetic branch controls restoration and relaxation [16]. Sympathetic activity can be indexed by skin conductance level (SCL) [17,18], and parasympathetic activity can be indexed by the high frequency part of the power spectral density of heart rate variability (HF HRV) [19,20].

Psychological stress can be elicited by factors such as failure to achieve and marital problems, psychological stress also often has physiological consequences [21]. In the laboratory, psychological stress is commonly induced by mental arithmetic and speeded Stroop tasks [22,23].

The purpose of the present study was to induce psychological stress and compare effects of different sound conditions on the rate of physiological recovery. The sound conditions were chosen so that a pleasant natural sound environment was compared with three less pleasant urban sound environments dominated by noise. To study effects of sound pressure level on physiological recovery, the urban sound conditions had higher, equal, or lower average sound pressure levels than the nature sound. Two measures of physiological stress were used: SCL as an index of sympathetic activity and HF HRV as an index of parasympathetic activity. Physiological recovery is associated with a decrease in sympathetic activation (*i.e.*, SCL decreases) and an increase in parasympathetic activation (*i.e.*, HF HRV increases). Because physiological stress recovery should be faster during exposure to pleasant than to unpleasant sounds, we hypothesized that (a) SCL should decrease faster and (b) HF HRV increase faster during pleasant nature sound than during less pleasant noise.

## 2. Methods

### 2.1. Participants

Forty university students participated in the experiment (24 women and 18 men, mean age = 27 years). All participants had hearing thresholds lower than 25 dB in their best ear, for all tested frequencies (0.125, 0.5, 1, 2, 3, 4, and 6 kHz, Interacoustics Diagnostic Audiometer, model AD226). The listeners received course credit for their participation. Electrocardiogram data were missing from three participants (1 man, 2 women) due to electrode failure.

### 2.2. Experimental Design

**Figure 1.**
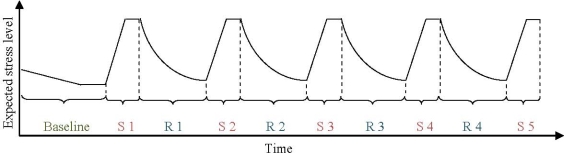
Experimental design with experiment duration on x-axis and expected stress level on y-axis (S = stress test; R = recovery period for each experimental sound condition).

The experiment consisted of three different parts: (1) One 5-min quiet baseline period, (2) five 2-min periods of testing (“stressor”), and (3) four 4-min periods of relaxation (“recovery”) during exposure to one of four experimental sounds. Figure 1 illustrates the experiment schematically. Total time for the experiment was approximately 35 minutes.

A 4 × 4 mixed design was used, with sound during relaxation as within subject variable and presentation order of the four sounds as between subject variable (the sounds are described in detail below). The participants were randomly assigned to one of four orders of experimental sounds, using a Latin square matrix.

### 2.3. Stressor

The stressor was a two minute speeded mental arithmetic task (henceforth “the stress test”). The task was to decide, within 3 s, whether a displayed equation was correct or false. The participants answered by pressing one of two keys on a numeric keyboard. Their responses were evaluated as either “correct”, “false” or “too late” (if later than 3 s). Feedback was presented on the screen (correct, false or too late) and through earphones with a specific sound for each type of feedback. The equations consisted of simple arithmetic operations, such as ‘543−345 = 193’. The first two terms were integers between 2 and 999, and the result of the equation was a positive integer below 1,000 which either was correct or false (correct answer +/− 1–3). The operator could either be addition, subtraction, division or multiplication. Each operator had 250 equations in a database, half correct and half false. Overall performance (percent correct) was continuously updated and displayed to the participants in the upper left corner of the screen.

### 2.4. Experimental Sounds

During each of the four recovery periods, participants were exposed to either a nature sound or a noise. The sound pressure levels of the noises were higher, equal or lower than the sound pressure level of the nature sound. The experimental sounds were selected from a large database of binaural recordings of environmental sounds. The nature sound was chosen to be more pleasant than the three noises, including the ambient noise of lower sound pressure level. The four experimental sounds are described below.

1. *Nature sound*. A mixture of sounds from a fountain and tweeting birds. The average sound pressure level was set to 50 dB (*L*_Aeq,4min_).
2. *High noise*. Road traffic noise recorded close to a densely trafficked road. The average sound pressure level was set to 80 dB (*L*_Aeq,4min_).
3. *Low noise*. The same noise as (2), but set to a lower average sound pressure level, 50 dB (*L*_Aeq,4min_).
4. *Ambient noi*se. A recording from a quiet backyard, with a constant low level ambient noise, mainly caused by ventilation systems of the buildings surrounding the yard. The average sound pressure level was set to 40 dB (*L*_Aeq,4min_).

### 2.5. Physiological Measures

For SCL measurement two electrodes were fitted by the experiment leader to the hypothenar eminence of the non dominant hand. The SCL was measured as the conductance between the two electrodes.

HRV measurements were derived from the electrocardiogram (ECG). Three electrodes were applied by the participant themselves under supervision of the experimenter. The first electrode was positioned five centimeters to the right of the middle of the upper sternum and the other two on the left and right side of the stomach, just below the ribcage. HRV was calculated according to the procedure described by Berntson and by Hejjel & Kelleny [24,25].

### 2.6. Procedure

Participants were informed that the goal of the experiment was to study physiological reactions during a stressful task and that there would be sound presentations during the four minute pauses. The participants were tested individually. They were first asked to wash their hands and were then seated in a soundproof room, where they were given a written description of the experiment. After the participants had given their consent to participate, electrodes were fitted to their bodies. They were then asked to put on a pair of headphones and one trial version of the stress test was completed in order to check the equipment.

During the baseline period, the participants were asked to relax in silence. At the end of the period a prerecorded female voice reminded them that the first stress test was about to begin. After the stress test, the female voice instructed the participants to relax and one of the four experimental sounds was presented. This switch between stress test and recovery was repeated three more times (see Figure 1).

After the experiment, participants listened to the four experimental sounds one more time and assessed the sound’s pleasantness, eventfulness, and familiarity on three bipolar category scales. These attributes have been suggested as basic perceptual dimensions of sound environments [26]. Finally, the participants’ thresholds of hearing were tested. Participants were informed only after the experiment about the true purpose of the study (*i.e.*, our interest in the sounds). The study was conducted in accordance with regional ethical guidelines.

### 2.7. Equipment

The sounds were recorded with a binaural head and torso simulator Brüel & Kjær type 4100, with two microphones type 4190 and two pre-amplifiers type 2669, one conditioning amplifier NEXUS Brüel & Kjær type 2690 A 0S4 and a calibrator Brüel & Kjær type 4231 plus adapter model 0887. A portable computer Dolch NPAC-Plus P111 with a 6-channel LynxTwo sound card stored the recordings with 24 bit resolution and 48 kHz sampling frequency using Sound Forge 7. Editing and mixing was performed in the same program. In the soundproof room, the signal was fed into a digital filter and D/A-converter, Rane RPM 26z, and was then presented through Sennheiser HD 600 headphones. The whole listening system was calibrated using a pink-noise signal, measured at the point of the listener’s ear. The frequency response of the whole listening system was flat within 2 dB, 1/3-octave-band levels, 25−16,000 Hz.

The physiological data were recorded with a Biopac System MP100 at 1000 Hz. SCL was measured with a Biopac GSR100C amplifier and EDA isotonic gel electrodes and ECG was measured with a Biopac ECG100C amplifier and Red Dot™ Ag/AgCl solid gel electrodes.

Both programming and presentation of the mental arithmetic stress task was conducted in Matlab 6.5. The physiological data were analyzed in Matlab, while the HRV power density spectrum (PDS) was computed in ACQ Knowledge 3.91. Statistical analyses were conducted in SPSS 16.

## 3. Results

### 3.1. Perceptual Assessment of Experimental Sounds

**Figure 2.**
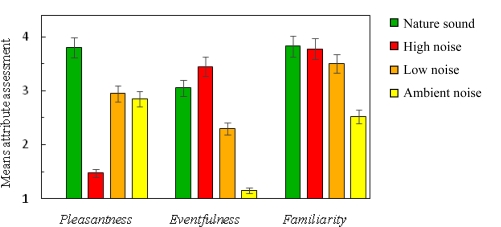
Mean values of perceptual attributes for the nature sound and the high, low and ambient noises. Error bars represent the standard error of the mean.

The perceptual assessment of the sounds showed that the nature sound was perceived as more pleasant than the noises (Figure 2). This confirms that the selection of sounds was successful, as the goal was to find a nature sound that was more pleasant than any of the noises. The low noise and the ambient noise were similar in perceived pleasantness whereas the high noise sound was rated as the least pleasant sound. The perceptual evaluation also showed that the high noise was perceived as more eventful than the other sounds. The ambient noise was the least eventful and also the least familiar sound, probably because it contained no distinct sound sources and therefore was perceived as an undifferentiated background noise.

### 3.2. Physiological Measures

**Figure 3.**
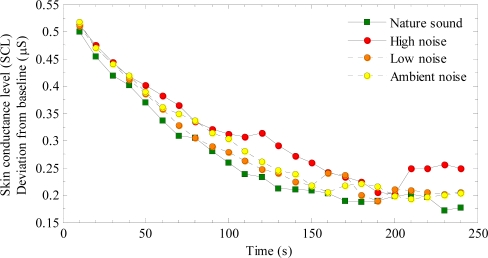
Baseline corrected skin conductance level (SCL) as a function of time, shown separately for recovery during exposure to nature, high noise, low noise and ambient sound.

*Skin conductance level (SCL)*: The mean of successive 10 second periods were computed for the recovery periods after each stress test. The mean of seconds 150–270 of the baseline period was used as the baseline measure. Figure 3 shows baseline corrected SCL values over time, and Table 1 shows descriptive statistics of these data.

Figure 3 suggests that although SCL immediately after the stressor was similar for the different conditions, recovery was faster during exposure to the nature sound than to the three noise conditions. The ambient and low noise had the second fastest, and high noise the slowest recovery. A slight upswing during the last 50 seconds of the recovery period was seen for SCL recovery during the high noise, possibly reflecting an increased arousal due to prolonged exposure to the unpleasant noise. In a 4 × 4 mixed ANCOVA, the mean SCL for each participant during the recovery period was used as the dependent variable, sound as a within-subjects variable, and presentation order as a between-subjects variable. The baseline measure was included in the analysis as covariate [27].

The ANCOVA showed an interaction between presentation order and sound (F_9,105_ = 6.851, p = 0.001). This effect reflected general SCL-increase during the experiment. The main effect of sound was significant (F_3,105_ = 2.731, p = 0.048). Pairwise comparisons (t-tests) showed that mean SCL was lower for Nature than High noise (p = 0.045); however, the differences between Nature and the other two noise conditions did not reach significance (p > 0.05).

**Figure 4.**
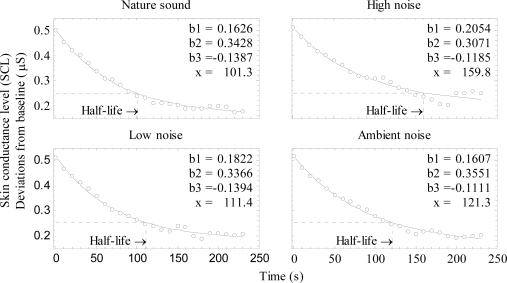
Skin conductance level (SCL) as a function of time, shown separately for the four sounds. Curves were fitted to the group data. Constants of Equation 1 and half life value (x) are indicated in each diagram.

In an ANCOVA of sound (4) × time (24, the successive 10 second periods) with baseline as covariate, the interaction between sound and time was significant (F_69,2622_ = 1.34, p = 0.034). This finding suggests that recovery time for SCL differed among the sound conditions. As Figure 3 suggests that polynomial trends (e.g., linear, quadratic) in an ANCOVA would not describe the recovery functions well, a non-linear regression analysis was performed to obtain point estimates of recovery time. To that end, we fitted an exponential function (Equation 1) to the mean SCL data shown in Figure 3:

(1) $$y=b_{1}+b_{2}e^{b_{3}x},$$

where *y* is baseline corrected SCL, *x* is time (in seconds) and b_1_, b_2_ and b_3_ are constants. Figure 4 shows the fitted functions for the four experimental sounds. The fit, R^2^, for the nature sound, low noise and ambient noise was > 0.99, it was slightly lower for the high noise, R^2^ = 0.96. RMS-error for the nature, high noise, ambient and low noise sound was 0.0088, 0.017, 0.0090 and 0.0097 μS, respectively. The half life recovery was calculated using Equation 1, by solving for *x* at the point where SCL had been reduced by half, compared with its value at *x* = 0 (see dotted line in Figure 4). The high noise had the longest half life of 159.8 s, the half life of the other three were 121.3 s for ambient noise, low noise 111.4 s and nature sound 101.3 s. Reliable statistical testing of individual half life values was not possible, since the estimated constants in several cases generated complex numbers, that resulted in missing data when half life values were calculated.

*Heart rate variability (HRV)*: We found no consistent effect of type of sound on HF HRV. Average HRV values were not higher for nature sound than for the other sounds, and HF HRV for the high noise was not substantially lower than for the other sounds. The HF HRV data for each participant and recovery condition were tested in a 4x4 ANCOVA, with type of sound as within subject variable and presentation order as between-subject variable. The baseline measure was included in the analysis as covariate [27]. Neither type of sound, presentation order nor their interaction was statistically significant (p > 0.05).

## 4. Discussion

The main purpose of this study was to test whether physiological stress recovery is faster during exposure to pleasant nature sounds than to noise. Figure 3 suggests that mean SCL during the nature sound was lower than for the noises. Although this difference was statistically significant only between the nature sound and the high noise, detailed analyses of the recovery functions showed that half-life SCL recovery was 9−37% faster during the nature sound than during the noises. These results suggest a faster recovery of the sympathetic nervous system [16,17] during the nature sound. Because HF HRV showed no effects of experimental sounds, this null finding suggests that the parasympathetic activation may be less affected by sound during recovery.

The present results suggest that recovery from sympathetic arousal is affected by type of sound (nature sound *versus* noise). Recovery was faster during the nature sound (50 dBA) compared with the noises, including the low noise (50 dBA) and the ambient noise (40 dBA). The mechanisms behind the faster recovery could be related to positive emotions (pleasantness), evoked by the nature sound as suggested by previous research using non audio film stimuli [9]. Other perceptual attributes may also influence recovery. The Ambient noise was perceived as less familiar than the other sounds (Figure 2), presumably because it contained no identifiable sources. One may speculate that this lack of information might have caused an increased mental activity and thereby an increased SCL, compared with the nature sound (cf. [28]). An effect of sound pressure level can be seen in the difference between high and low noise, this difference is in line with previous psychoacoustic research [12] and is not a surprising considering the large difference (30 dBA) in sound pressure level.

The results from SCL are consistent with those of Ulrich *et al.* [6], who found a faster decrease in SCL after audio-visual exposure to natural compared with urban environments. Ulrich *et al.* found a similar effect on heart period. This disagrees with our HF HRV results. An explanation might be that heart period is influenced by both sympathetic and parasympathetic activity [6], whereas our measure, the HF part of the HRV, only is related to parasympathetic activity [17,19], the effects found on heart period reflects the influence of the sympathetic rather than the parasympathetic system. The lack of effects on HF HRV in our study suggests that sound exposure during recovery may not have a strong influence on parasympathetic activity, at least not with the exposures and the physiologic indexes used in the current experiment.

Our study used a small set of environmental sounds of short duration, which limits the generalizability of the results. The nature sound used in the experiment had a relatively high sound pressure level (50 dBA). The effect of natural sound environments on stress recovery may be greater in situations with longer exposure times and with lower sound pressure levels commonly found in recreational and rural areas outside cities. In city parks and other urban outdoor areas, the sound environment is typically a mix of sound from nature sounds and traffic noise. Based on the present results, it seems plausible to speculate that recovery from sympathetic activation in such areas would be less effective than in areas undisturbed by noise.

## 5. Conclusions

The present results suggest that after psychological stress, physiological recovery of sympathetic activation is faster during exposure to pleasant nature sounds than to less pleasant noise of lower, similar, or higher sound pressure level.

## Figures and Tables

**Table 1.** The mean, median, max, min and standard deviation (SD) of participants (n = 40) for SCL and HF HRV, baseline measures were computed from second 150–270, recovery measures were computed over the whole recovery period.

Period	Sound	Statistic (N = 40)				
		Mean	Median	Max	Min	SD
	*Skin conductance level (SCL), μS*					
Baseline	Silence	0.55	0.53	2.01	0.09	0.39
Recovery	Nature	0.82	0.84	1.84	0.25	0.36
	High Noise	0.87	0.90	1.49	0.25	0.35
	Low noise	0.84	0.86	1.69	0.17	0.39
	Ambient	0.85	0.84	1.80	0.28	0.38
	*High frequency heart rate variability (HF HRV), ms^2^/Hz*					
Baseline	Silence	0.60	0.55	1.08	0.29	0.21
Recovery	Nature	0.59	0.58	0.91	0.27	0.18
	High Noise	0.60	0.57	1.01	0.31	0.20
	Low noise	0.60	0.53	1.07	0.25	0.21
	Ambient	0.61	0.57	1.04	0.32	0.19

## Abbreviations:

HF HRV: High frequency heart rate variability
SCL: Skin conductance level
